# Maternal and Neonatal Outcomes of Water Birth Compared with Conventional Vaginal Delivery: A Five-Year Retrospective Cohort Study from Southeastern Europe

**DOI:** 10.3390/life16040661

**Published:** 2026-04-13

**Authors:** Dragos Brezeanu, Ana-Maria Brezeanu, Simona Stase, Vlad Tica

**Affiliations:** 1 6th Department, Faculty of Medicine, Ovidius University of Constanta, 900527 Constanta, Romania; brezeanudragos@gmail.com (D.B.); stase.simona.10d@gmail.com (S.S.); vtica@eeirh.org (V.T.); 2 Armonia Hospital, 900500 Constanta, Romania; 3 Romanian Academy of Scientists, 050444 Bucharest, Romania

**Keywords:** water birth, land birth, vaginal birth, cesarean section, maternal outcomes, neonatal outcomes, episiotomy, physiological childbirth

## Abstract

Background: Water birth has gained increasing attention as an alternative childbirth method intended to promote maternal comfort and physiological labor while potentially reducing obstetric interventions. However, evidence regarding its maternal and neonatal safety compared with conventional delivery approaches remains heterogeneous. This study aimed to evaluate maternal and neonatal outcomes associated with water birth compared with conventional spontaneous vaginal delivery in a secondary obstetric center. By focusing on vaginal births, the study evaluates the specific impact of water immersion on perineal integrity and neonatal transition. Methods: We conducted a retrospective cohort study including 3747 deliveries recorded at a tertiary maternity unit over a five-year period. Among these, 692 births occurred in water (water birth group) and 561 were conventional vaginal deliveries (land birth group), both managed under a standardized institutional protocol. Maternal characteristics, obstetric outcomes, and neonatal parameters were extracted from medical records and compared between the two cohorts. Primary outcomes included rates of episiotomy, perineal trauma and neonatal indicators such as Apgar score. Statistical analyses were performed to assess differences between groups using appropriate comparative tests. Results: Water birth was associated with a significantly lower rate of episiotomy compared with land birth (5.06% vs. 13.72%, OR 0.33, 95% CI 0.22–0.50, *p* < 0.001). Neonatal outcomes, including Apgar scores and NICU admissions, did not differ significantly between the two cohorts. Conclusions: In this retrospective cohort, water birth among selected low-risk pregnancies was associated with reduced obstetric intervention, particularly episiotomy, without evidence of adverse neonatal outcomes. These findings suggest that water birth may represent a safe and feasible option in carefully selected low-risk pregnancies when strict clinical protocols are applied.

## 1. Introduction

Childbirth represents one of the most significant physiological processes in human life, yet modern obstetric practice has increasingly shifted toward highly medicalized models of care [1,2]. In recent decades, the global rise in obstetric interventions—including induction of labor, instrumental delivery, and cesarean section—has generated growing debate regarding the balance between necessary medical intervention and the preservation of physiological childbirth [3,4]. In response to this challenge, increasing attention has been directed toward strategies that support the natural mechanisms of labor while maintaining maternal and neonatal safety [5]. Among these approaches, water immersion during labor and water birth have emerged as increasingly popular alternatives in contemporary obstetric care [6].

The concept of water immersion during labor is based on the premise that a warm aquatic environment may facilitate the physiological progression of childbirth [7]. Immersion in water provides buoyancy and relaxation of the musculoskeletal system, allowing greater maternal mobility and reducing gravitational pressure on the pelvic structures [8]. These effects may contribute to more efficient uterine contractions and facilitate fetal descent through the birth canal. In addition, warm water immersion has been associated with reduced maternal stress responses, potentially mediated by decreased catecholamine levels and increased endorphin release [6]. Such physiological changes may improve maternal comfort, reduce pain perception, and promote a more positive labor experience.

Water immersion has therefore been proposed as a non-pharmacological method of pain relief during labor [9]. Several observational studies and clinical reports have suggested that immersion in water may reduce the need for pharmacological analgesia and improve maternal satisfaction with the birth experience [7,10]. The increased sense of autonomy and the possibility of a less medicalized birth environment have contributed to the growing popularity of water birth, particularly among birthing people seeking physiological childbirth [11]. Consequently, an increasing number of maternity units worldwide have begun to integrate water birth into their obstetric services [11,12].

Despite these potential benefits, the safety of water birth remains a subject of ongoing debate within the obstetric community. Concerns have historically focused on possible neonatal risks, including water aspiration, infection, thermoregulation difficulties, or delayed neonatal adaptation immediately after birth [13,14]. Early reports describing rare adverse neonatal events contributed to a cautious approach among some professional organizations and maternity units [15,16]. However, more recent studies and systematic reviews have generally reported comparable neonatal outcomes between water birth and land birth when strict selection criteria and appropriate clinical protocols are applied [5,17]. In most contemporary studies, neonatal parameters such as Apgar score, neonatal resuscitation, and short-term neonatal morbidity appear similar between births occurring in water and those occurring on land [18].

Beyond neonatal safety, the potential impact of water birth on maternal outcomes represents an important area of investigation. Several studies have suggested that water birth may be associated with reduced rates of obstetric interventions, particularly episiotomy [19,20]. The increased elasticity of perineal tissues in a warm aquatic environment, combined with slower and more controlled delivery of the fetal head, may contribute to a lower incidence of perineal trauma [21]. In addition, the ability of the birthing person to adopt more comfortable and physiologically favorable positions during water immersion may facilitate spontaneous vaginal birth and reduce the need for instrumental assistance [22]. These factors are particularly relevant in the context of ongoing efforts to reduce unnecessary obstetric interventions and promote physiological childbirth [23].

Globally, the rate of cesarean section has risen substantially over the past decades, frequently exceeding the levels recommended by international health organizations [24,25]. Although cesarean section remains a life-saving procedure when medically indicated, excessive use of this intervention has been associated with increased maternal morbidity, prolonged recovery, and potential complications in subsequent pregnancies [26]. Consequently, identifying safe strategies that support vaginal birth while maintaining favorable maternal and neonatal outcomes has become an important priority in contemporary obstetrics [24,27]. In this context, water birth has increasingly been considered a potential approach to encourage physiological labor and reduce intervention rates in carefully selected pregnancies [15,16].

In Southeastern Europe, however, access to water birth remains limited and published clinical data from this region are scarce [28]. In Romania, most maternity units continue to follow conventional obstetric delivery models, and water birth is not widely available within the public healthcare system [29,30]. Despite the growing international literature on water birth, evidence from Eastern and Southeastern Europe remains scarce, particularly from structured hospital-based programs. This gap limits the generalizability of existing evidence to healthcare systems with different obstetric practices.

Armonia Private Hospital, located in Southeastern Romania, represents one of the few maternity centers in the region offering birthing people the possibility of giving birth in water within a supervised clinical setting. Over recent years, this center has accumulated considerable clinical experience with water birth, providing an opportunity to evaluate maternal and neonatal outcomes associated with this birth method in a relatively large cohort.

Given the ongoing debate regarding the safety and clinical implications of water birth, additional evidence derived from real-world clinical practice may contribute to a better understanding of its role in modern obstetric care. Therefore, the aim of the present study was to perform a comparative analysis between water birth and land birth in a five-year retrospective cohort conducted at Armonia Private Hospital between 2020 and 2024. This approach was designed to isolate the effects of water immersion on labor progression and perineal outcomes while excluding the confounding surgical variables associated with cesarean sections. By analyzing birth outcomes in this unique regional cohort, the present study aims to contribute further evidence regarding the role of water birth in contemporary obstetrics and its potential impact on maternal and neonatal health.

## 2. Materials and Methods

### 2.1. Study Design and Setting

This study was designed as a retrospective observational cohort study conducted at Armonia Private Hospital, Constanța, Romania. Armonia is a secondary-level private maternity hospital located in Southeastern Romania and represents the only maternity center in the region offering the possibility of water birth within a supervised clinical setting.

Of the 3747 deliveries included, 1253 (33.5%) were vaginal births, subsequently subdivided into two comparative cohorts: the water birth group (*n* = 692) and the land birth group (*n* = 561). Cesarean sections (*n* = 2494) were retained in the overall cohort description but excluded from the primary comparative analysis of intrapartum and perineal outcomes, as the focus of this study was the specific effect of water immersion during vaginal birth.

The study covered a five-year period, between 1 January 2020 and 31 December 2024, during which all deliveries recorded in the institutional electronic obstetric database were reviewed. As a secondary-level maternity hospital, the institution manages pregnancies from 34 weeks of gestation onward, and therefore only deliveries occurring at ≥34 weeks of gestation were included in the present analysis.

The primary objective of the study was to evaluate and compare maternal and neonatal outcomes associated with two modes of birth: water birth and conventional spontaneous vaginal delivery. While cesarean sections were recorded in the hospital’s database during the study period, they were excluded from the comparative analysis of birth outcomes (such as perineal trauma and labor duration) to ensure a homogenous study population and to focus specifically on the impact of water immersion on the physiological birth process. Neonatal intensive care unit (NICU) admission was recorded as a secondary neonatal outcome.

### 2.2. Data Collection and Study Population

Data were obtained from the hospital’s electronic obstetric registry, in which maternal and neonatal parameters are routinely documented immediately after birth as part of standard clinical practice. The database contains detailed information regarding birth characteristics, neonatal parameters, and selected maternal outcomes.

**Figure 1:**
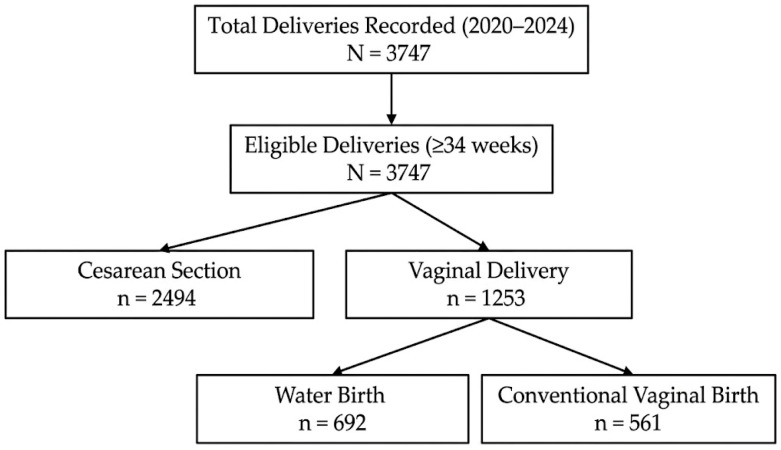
STROBE flow diagram of the study population. All deliveries recorded at Armonia Private Hospital between January 2020 and December 2024 were included in the analysis (*n* = 3747). Births were categorized according to mode of birth into cesarean section and vaginal birth. Vaginal births were further subdivided into land birth and water birth. Cesarean sections were retained in the overall cohort description but excluded from the primary intrapartum outcome comparison, which focused on the effect of water immersion during vaginal birth.

All deliveries recorded during the study interval were screened and included in the analysis. Because the institutional database systematically records every delivery performed at the hospital, no cases were excluded, resulting in a final cohort of 3747 deliveries. No formal sample size calculation was performed because the study included the entire cohort of deliveries recorded at the institution during the study period. The study was reported in accordance with the STROBE guidelines for observational studies as shown in Figure 1.

Deliveries were initially classified according to mode of birth as either cesarean section or vaginal delivery. Vaginal births were subsequently subdivided into land birth and water birth, based on documentation in the delivery registry.

Water birth was defined as a vaginal birth completed in water within the hospital’s dedicated water birth facility, under continuous supervision by a specialist obstetrician and a certified midwife. In Romania, all births, including water births, are attended by a specialist obstetrician supported by midwifery staff, in accordance with national obstetric practice. This differs from midwifery-led water birth models practiced in some other countries, where obstetric input is sought upon request. This classification allowed the comparison of outcomes between the three predefined delivery groups.

### 2.3. Eligibility Criteria for Water Birth

Water birth was offered exclusively to birthing people with carefully selected low-risk pregnancies, according to the institutional protocol of the maternity unit. Eligibility criteria included: singleton pregnancy, cephalic presentation, gestational age between 37 and 41 weeks, spontaneous onset of labor, reassuring fetal heart rate monitoring, and absence of significant maternal or fetal complications.

Women were excluded from water birth if any of the following conditions were present: multiple pregnancy, non-cephalic presentation, preterm or post-term pregnancy, hypertensive disorders of pregnancy (including preeclampsia), gestational or pregestational diabetes requiring pharmacological treatment, suspected fetal growth restriction, intrapartum fetal distress, meconium-stained amniotic fluid, maternal fever, or any condition requiring continuous electronic fetal monitoring or immediate obstetric intervention.

These criteria were applied to ensure that water birth was reserved for birthing people with uncomplicated pregnancies and a low predicted risk of adverse maternal or neonatal outcomes.

Each medical record was subsequently individually reviewed by two independent investigators (A.M.B. and D.B.) to confirm eligibility according to the inclusion and exclusion criteria. Discrepancies were resolved through joint re-examination and, when necessary, consultation with a third senior reviewer (V.T.). This dual-review process minimized classification bias and ensured uniform application of definitions across all cases.

### 2.4. Outcome Measures

The primary neonatal outcomes evaluated in this study included birth weight and Apgar score, both routinely recorded in the birth documentation.

Birth weight was analyzed as a continuous variable and, where appropriate, categorized according to commonly accepted clinical thresholds, including low birth weight (<2500 g) and macrosomia (>4000 g).

In addition to neonatal parameters, selected maternal outcomes associated with mode of delivery were evaluated, including obstetric interventions such as episiotomy, when recorded in the institutional database.

### 2.5. Statistical Analysis

Statistical analysis was performed using IBM SPSS Statistics version 29 (Faculty Pack, IBM Corp., Armonk, NY, USA).

Descriptive statistics were used to summarize the characteristics of the study population. Continuous variables were expressed as means with standard deviations, while categorical variables were presented as absolute frequencies and percentages. Although cesarean sections were included in the overall cohort description, the primary comparative analysis of maternal and neonatal outcomes focused on vaginal births, specifically comparing the water birth cohort with the land birth cohort.

Comparative analyses were conducted to evaluate differences between birth modes. Continuous variables were analyzed using the independent-samples *t*-test or one-way analysis of variance (ANOVA), depending on the number of groups being compared. Categorical variables were analyzed using the chi-square test or Fisher’s exact test, as appropriate.

Two principal analytical comparisons were performed. First, cesarean section was compared with vaginal birth in order to evaluate overall neonatal outcomes. Second, within the subgroup of vaginal births, water birth was compared with land birth in order to assess potential differences associated with immersion during childbirth.

Continuous variables were expressed as mean ± standard deviation (SD) and compared using the independent-samples *t*-test. Categorical variables were compared using the chi-square (χ^2^) test. A *p*-value < 0.05 was considered statistically significant.

To account for the inherent selection bias of low-risk patients in the water birth group, we performed a multivariable logistic regression analysis. The models were adjusted for potential confounders, including maternal age, parity (nulliparous vs. multiparous), and gestational age. This allowed us to estimate adjusted Odds Ratios (aOR) for the primary maternal and neonatal outcomes.

### 2.6. Ethical Considerations

The study protocol was approved by the Ethics Committee of Armonia Private Hospital number 1/12 January 2026. The investigation was conducted in accordance with the principles outlined in the Declaration of Helsinki. Because the study consisted of a retrospective analysis of anonymized institutional data, the requirement for individual informed consent was waived. All data were de-identified prior to analysis to ensure patient confidentiality.

## 3. Results

### 3.1. Study Population and Annual Birth Distribution

During the five-year study period, between January 2020 and December 2024, a total of 3747 births occurring at a gestational age of 34 weeks or greater were recorded at Armonia Private Hospital and included in the analysis. Because the institutional obstetric database systematically records all births performed at the hospital, no cases were excluded from the final cohort. The annual number of births remained relatively stable throughout the study period, ranging from 712 births in 2020 to 782 births in 2022, with only minor fluctuations in subsequent years.

### 3.2. Distribution of Birth Modes and Cohort Composition

Among the 3747 births, 2494 (66.5%) were performed by cesarean section, while 1253 births (33.5%) were vaginal. Within the vaginal birth group, two distinct cohorts were identified for comparative analysis: the water birth cohort (n = 692; 55.2% of vaginal births; 18.5% of all births) and the land birth cohort—defined as out-of-water vaginal births—(n = 561; 44.8% of vaginal births; 15.0% of all births). These two cohorts form the basis of all primary comparative analyses reported in this study. The selection and classification of the study population according to mode of birth are illustrated in Figure 1 (STROBE flow diagram).

### 3.3. Baseline Maternal Characteristics

Baseline maternal characteristics were comparable between the water birth and land birth cohorts (Table 1). The mean maternal age was 30.73 ± 4.67 years in the water birth group and 30.20 ± 4.72 years in the land birth group. The overlapping 95% confidence intervals (30.38–31.08 and 29.81–30.59, respectively) and the nonsignificant *p*-value (*p* = 0.061) indicate that the two groups were homogeneous with respect to maternal age. Approximately 58% of birthing people were nulliparous in both groups, and the mean gestational age at birth was 39.1 weeks across the cohort, indicating that most pregnancies were carried to term. The comparability of baseline characteristics between groups supports the validity of the subsequent outcome comparisons.

### 3.4. Maternal and Neonatal Outcomes

Maternal and neonatal outcomes for the water birth and land birth cohorts are presented in a consolidated format in Table 2, which integrates descriptive statistics, unadjusted odds ratios, and adjusted odds ratios for all primary and secondary outcome measures.

The following sections describe the findings cohort by cohort for each primary and secondary outcome.

#### 3.4.1. Episiotomy

**Figure 2:**
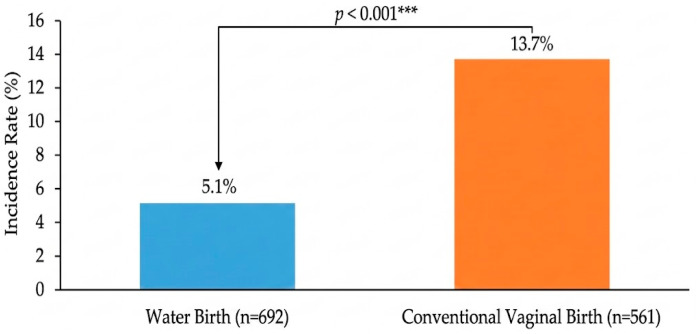
Incidence of episiotomy in water birth compared to land birth. The chart demonstrates a significant reduction in the rate of episiotomy among the water birth group (5.1%) compared to the land birth group (13.7%). This highly significant difference (*p* < 0.001, Chi-square test) supports a protective effect of warm water immersion on perineal integrity during the second stage of labor. Data presented as incidence percentages. *** *p* < 0.001.

A marked and highly significant difference was observed in the rate of episiotomy between the two vaginal birth groups. Episiotomy was performed in 13.72% of land births compared with only 5.06% of water births, representing a nearly three-fold reduction (OR 0.33, 95% CI 0.22–0.50, *p* < 0.001). As illustrated in Figure 2, the non-overlapping 95% confidence intervals for episiotomy rates (3.5–6.6% vs. 10.8–16.6%) confirm a statistically significant between-cohort difference. After adjustment for maternal age, parity, and gestational age, the difference remained statistically significant (aOR 0.44, 95% CI 0.29–0.68, *p* < 0.001).

#### 3.4.2. Perineal Tears and Postpartum Hemorrhage

In the water birth cohort, the rate of perineal tears (Grade I/II) was significantly lower compared with the land birth cohort, both before and after multivariable adjustment (aOR 0.44, 95% CI 0.29–0.68, *p* < 0.001). Regarding postpartum hemorrhage, rates were low in both cohorts and did not differ significantly between the water birth and land birth groups (aOR 0.74,95% CI 0.41–1.35, *p* = 0.320).

#### 3.4.3. Birth Weight

**Figure 3:**
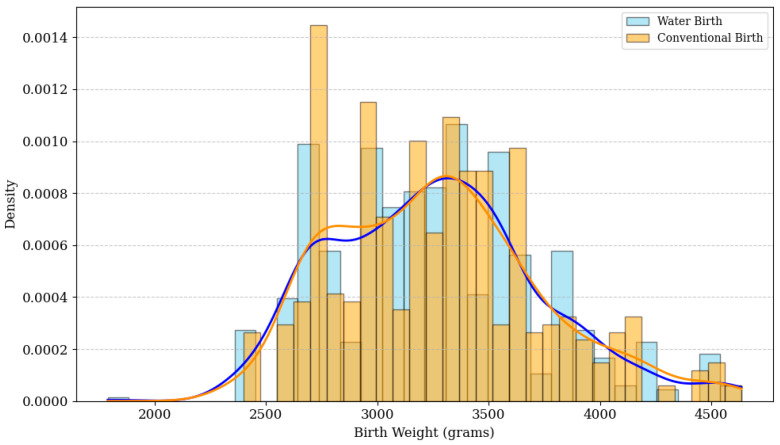
Birth weight distribution in water birth versus land birth cohorts (2020–2024). The histogram illustrates the frequency density distribution of birth weights (grams) for both cohorts. The overlapping kernel density estimate (KDE) curves and a *p*-value of 0.867 confirm no statistically significant difference in birth weight between groups. Blue bars and KDE curve: water birth cohort; orange bars and KDE curve: conventional (land) birth cohort.

Birth weight did not differ significantly between the water birth group (3261.7 ± 467.2 g) and the land birth group (3266.4 ± 463.6 g) (*p* = 0.867). The odds of low birth weight (<2500 g) were similar between groups (OR 0.92, 95% CI 0.38–2.22, *p* = 0.86). Figure 3 illustrates the overlapping birth weight density distributions for both cohorts, with virtually identical kernel density estimate curves.

#### 3.4.4. Apgar Score

**Figure 4:**
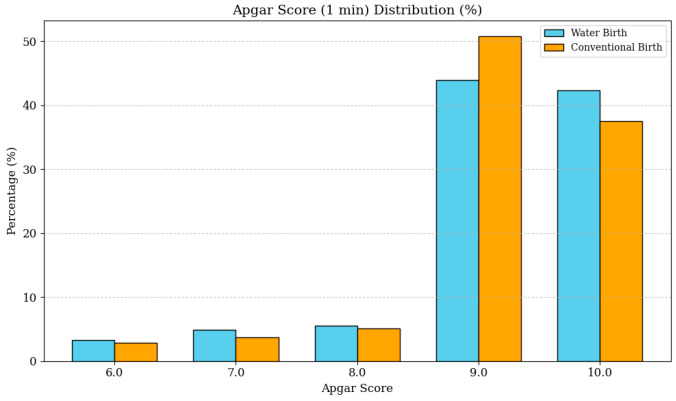
Apgar score (1 min) distribution in water birth versus land birth cohorts. The bar chart displays the percentage distribution of Apgar scores at 1 min for both groups. Most neonates in both cohorts achieved optimal scores of 9 and 10. The absence of significant difference (*p* = 0.971) supports the safety of water birth regarding immediate neonatal transition and respiratory adaptation.

Apgar scores at 1 min were similarly comparable between the water birth group (9.17 ± 0.97) and the land birth group (9.17 ± 0.90) (*p* = 0.971), indicating equivalent immediate neonatal adaptation in both groups. As shown in Figure 4, the vast majority of neonates in both cohorts achieved optimal Apgar scores of 9 or 10 at 1 min, with closely mirrored distributions across all score categories. The odds of a low Apgar score (<7 at 1 min) were comparable between groups (OR 0.94, 95% CI 0.35–2.53, *p* = 0.91). After multivariable adjustment, the odds of an Apgar score below 9 at 1 min were significantly lower in the water birth cohort compared with the land birth cohort (aOR 0.36, 95% CI 0.18–0.72, *p* = 0.004).

#### 3.4.5. NICU Admission

No neonates born in water required admission to the neonatal intensive care unit (NICU), whereas five neonates (0.9%) in the land birth group required NICU admission at birth (*p* = 0.062, Fisher’s Exact Test). This difference did not reach statistical significance. After multivariable adjustment, no significant difference in NICU admission rates was observed between cohorts (aOR 0.61, 95% CI 0.25–1.49, *p* = 0.275).

## 4. Discussion

The present study evaluated maternal and neonatal outcomes associated with water birth compared with land birth in a five-year retrospective cohort of 3747 births at a secondary-level private maternity hospital in Southeastern Romania. Water birth was associated with a significantly lower rate of episiotomy and perineal trauma, while neonatal outcomes were comparable between cohorts. These findings contribute real-world clinical evidence regarding the integration of water birth into modern obstetric practice when appropriate selection criteria and institutional protocols are applied [31,32]. With 692 water births, this cohort represents one of the largest reported clinical experiences with water birth in Southeastern Europe, providing evidence regarding the feasibility and safety of implementing water birth programs within structured obstetric settings in this region [33,34].

### 4.1. Neonatal Safety

A primary concern historically associated with water birth is the potential for compromised neonatal adaptation, including water aspiration, thermoregulatory instability, and increased infection risk [35,36,37]. The present findings do not support these concerns. In the water birth cohort, the odds of a low Apgar score at 1 min (<7) were comparable to those in the land birth cohort (OR 0.94, 95% CI 0.35–2.53, *p* = 0.91), and the odds of low birth weight showed no significant difference between cohorts (OR 0.92, 95% CI 0.38–2.22, *p* = 0.86). No neonates in the water birth cohort required NICU admission, compared with five (0.9%) in the land birth cohort, a difference that did not reach statistical significance (*p* = 0.062). These results are consistent with large-scale studies and systematic reviews, including Cochrane analyses, which conclude that water birth conducted in a controlled clinical setting for low-risk pregnancies does not increase neonatal morbidity [5,12,17]. Several observational studies and systematic reviews have reported similar Apgar scores, rates of neonatal resuscitation, and early neonatal morbidity between water births and land births [6,11,17].

Concerns regarding the risk of underwater gasping or water aspiration have been addressed by evidence demonstrating that the mammalian dive reflex effectively prevents such events in healthy neonates born in water [5,32]. Accumulating evidence from observational studies and systematic reviews has similarly not demonstrated an increased risk of neonatal infection when appropriate hygienic protocols are followed [6,11,12]. Large meta-analyses including tens of thousands of births have shown that water birth is not associated with increased maternal or neonatal infection rates [5,17,18,34]. In our cohort, although infection rates and neonatal temperature parameters were not analyzed as separate endpoints, the comparable Apgar scores between cohorts provide indirect evidence supporting neonatal safety in this setting. These findings are consistent with previous studies reporting no increase in neonatal morbidity, NICU admission, or early infectious complications in water births performed under controlled clinical conditions [6,12,17]. Furthermore, large-scale registry data have consistently shown that the risk of NICU admission does not differ significantly based on the mode of birth for low-risk pregnancies [5,18,34].

When water birth is conducted in a regulated hospital environment, strict protocols regarding water temperature control—maintained between 37.0 °C and 37.5 °C to preserve the newborn’s dive reflex while preventing hyperthermia or thermal shock [38,39]—continuous fetal monitoring, and immediate skin-to-skin contact after birth help maintain neonatal thermal stability and minimize infection risk [15,19,31]. These measures align with international guidelines emphasizing that immediate skin-to-skin contact in a warm environment is a key factor in successful neonatal thermoregulation [23,40]. The neonatal safety profile in our cohort was maintained after multivariable adjustment, reinforcing the safety of this method in a controlled clinical setting.

### 4.2. Perineal Outcomes and Episiotomy in the Romanian Context

The water birth cohort demonstrated a nearly three-fold reduction in episiotomy rates compared with the land birth cohort (5.06% vs. 13.72%; OR 0.33, 95% CI 0.22–0.50, *p* < 0.001). After multivariable adjustment for maternal age, parity, and gestational age, this difference remained statistically significant (aOR 0.44, 95% CI 0.29–0.68, *p* < 0.001), confirming that the benefit is independent of the lower-risk profile of birthing people who selected water birth. The rate of perineal tears (Grade I/II) was similarly reduced in the water birth cohort after adjustment (aOR 0.44, 95% CI 0.29–0.68, *p* < 0.001), while postpartum hemorrhage rates did not differ significantly between cohorts (aOR 0.74, 95% CI 0.41–1.35, *p* = 0.320). These findings are consistent with the growing body of international evidence that warm water immersion is associated with reduced perineal intervention during labor [40,41,42]. Reducing unnecessary perineal trauma has important implications for postpartum recovery, maternal comfort, and long-term pelvic floor health [42,43,44].

Several physiological mechanisms may underlie these perineal outcomes. Warm water immersion promotes relaxation of the pelvic floor musculature and increases tissue elasticity, facilitating gradual stretching of the perineum during fetal head expulsion [11,19,21]. Buoyancy within the aquatic environment allows greater mobility and the adoption of physiologically favorable birthing positions, reducing mechanical stress on perineal structures and enabling a more controlled birth of the fetal head [7,22]. Together, these effects may reduce the clinical indication for episiotomy in water birth settings, supporting the role of water immersion as a non-pharmacological strategy for perineal protection [9,10]. By providing a more supportive and less stressful environment during labor, immersion in warm water may also contribute to improved maternal comfort, reduced pain perception, and more efficient progression of labor, further supporting maternal autonomy and birth experience [1,3,7,19].

It is important to contextualize these findings within the specific Romanian obstetric landscape. Romania has one of the highest episiotomy rates in Europe, with national estimates consistently exceeding 60–70% of vaginal births in public hospital settings [29,30]. Against this national background, the land birth episiotomy rate of 13.72% observed at our institution is substantially lower than the national average, likely reflecting the selective, low-risk profile of the private maternity setting and institutional efforts to reduce routine episiotomy use. Nevertheless, the 5.06% episiotomy rate in the water birth cohort remains non-negligible and warrants explanation.

Episiotomy during water birth at our institution is performed when clinically indicated, typically for signs of imminent severe perineal laceration or fetal compromise requiring expedited birth. In such cases, the birthing person is repositioned to a semi-recumbent or lateral position at or above the water surface, allowing the birth attendant direct visual access to the perineum. The procedure is performed under local anesthetic infiltration following the same clinical protocol applied in land births. The aquatic environment does not preclude adequate visualization of the perineum when appropriate positioning is adopted, and the procedure does not differ substantively from standard obstetric practice in this respect [19,31,35]. This approach ensures that the clinical safety standards associated with episiotomy are maintained regardless of birth environment.

Beyond the intrapartum benefits, reduced perineal trauma associated with water birth may also create a more favorable biological environment for postpartum tissue repair and recovery, a perspective that warrants further investigation [38,39]. Emerging evidence suggests that the preservation of tissue integrity creates a more favorable local microenvironment for epithelial regeneration, angiogenesis, and collagen remodeling [38,39]. In this context, regenerative approaches such as platelet-rich plasma (PRP) application and lactic acid-based topical formulations have been investigated as strategies to further support postpartum perineal healing, with promising results regarding wound recovery and maternal quality of life [40,41,42,43]. The combination of reduced intrapartum trauma associated with water birth and these emerging postpartum therapies may represent a synergistic approach to optimizing maternal recovery [7,39,44,45].

### 4.3. Cesarean Section Rates: Institutional and National Context

The overall cesarean section rate of 66.5% observed in this cohort substantially exceeds both the WHO-recommended threshold and the European average, and warrants specific discussion [23,24]. This finding must be interpreted within the specific institutional and sociodemographic context of the study population. Romania has among the highest cesarean section rates in the European Union, with national figures consistently reported between 45% and 50% across all settings and substantially higher in the private sector [28,29]. This trend reflects a complex interplay of factors including birthing person preference, medicolegal considerations, limited access to evidence-based intrapartum care in some regions, and a historical under-emphasis on physiological birth support within the Romanian obstetric system [29,30]. The global rise in cesarean section rates has become one of the most significant challenges in contemporary obstetric care [24,33], with rates consistently surpassing recommended thresholds worldwide [27]. While cesarean section remains an essential life-saving intervention when medically indicated, its excessive use has been associated with increased maternal morbidity, longer postpartum recovery, and potential complications in future pregnancies [25,27,30]. Consequently, contemporary obstetrics faces the challenge of balancing necessary medical intervention with the preservation of physiological birth [1,3,33], and strategies that support spontaneous vaginal birth and reduce unnecessary interventions are gaining increasing attention [2,10].

At our institution, the elevated cesarean rate likely reflects the specific patient profile of a private secondary-level maternity facility, including higher rates of maternal request cesarean birth, older mean maternal age, a greater proportion of pregnancies achieved through assisted reproductive technologies, and clinical practice patterns that may favor surgical birth in borderline clinical situations [25,29,35]. As a secondary-level center accepting births from 34 weeks of gestation, the institution may also receive a proportion of referred higher-risk pregnancies carrying obstetric indications for cesarean birth. This high-intervention background does not invalidate the comparative analysis between water birth and land birth cohorts—which was conducted exclusively within the vaginal birth subgroup—but does limit the generalizability of the overall cohort characteristics to other clinical settings [1,3].

An important limitation of the current study design is that the comparative cohorts were established exclusively within the vaginal birth subgroup, after cesarean births had already been excluded. Consequently, this study cannot address whether the availability of water birth influenced the overall cesarean section rate at our institution, nor whether birthing people who opted for water birth had a different likelihood of intrapartum cesarean compared with those who did not. This represents a meaningful gap, as one potential benefit of water birth programs supporting physiological labor and potentially reducing intrapartum cesarean rates cannot be evaluated from the current data [21,34]. Future prospective studies should be designed to capture the full birth pathway, including intrapartum cesarean rates, among birthing people who initiate labor with the intention of water birth compared with those who do not [7,10,31].

### 4.4. Limitations

Several limitations of this study should be acknowledged. First, the retrospective, non-randomized design means that selection bias cannot be entirely excluded. The water birth cohort comprised exclusively low-risk pregnancies selected according to strict institutional eligibility criteria, while the land birth cohort included a broader clinical spectrum. Although multivariable adjustment was performed for maternal age, parity, and gestational age, other potential confounders, including maternal body mass index, labor duration, and use of labor induction were not available in the institutional database and could not be incorporated into the regression models.

Second, the 1 min Apgar score was the only neonatal adaptation parameter consistently recorded in the institutional database across the entire study period. The 5 min Apgar score, which carries greater prognostic significance for neonatal neurological outcome, was not systematically documented and could not be included in the analysis. Future studies should incorporate 5 min Apgar scores as a primary neonatal outcome measure.

Third, the study cohorts were established after excluding cesarean births, which precludes any analysis of whether water birth availability or uptake influenced the overall cesarean section rate at our institution. This limits the conclusions that can be drawn regarding the broader impact of water birth on the intrapartum intervention cascade. Furthermore, we do not have data on the number of labors initiated as low-risk spontaneous labors that subsequently required emergency cesarean section, nor on whether this rate differed between birthing people who labored in water versus on land. This represents a clinically important question that the current study design cannot address, and it should be a primary objective of future prospective studies designed to capture the complete birth pathway from labor onset to final mode of birth.

Fourth, the study was conducted at a single private maternity center with an established water birth protocol and experienced staff, which may limit the generalizability of findings to public or resource-limited settings without equivalent infrastructure or training.

Fifth, neonatal outcome assessment was limited to routinely recorded parameters such as birth weight, Apgar score, and NICU admission. Additional indicators such as cord blood gas analysis, early neonatal infection rates, and neonatal temperature measurements were not consistently available and could not be included.

Finally, long-term maternal outcomes including pelvic floor function, perineal healing trajectories, and patient-reported birth experience were not assessed. Future prospective multicenter studies with broader outcome measurement and multivariate adjustment for additional confounders are warranted to more definitively evaluate the role of water birth in contemporary obstetric care.

## 5. Conclusions

In this five-year cohort study, water birth was associated with comparable neonatal outcomes and significantly reduced rates of obstetric intervention, particularly episiotomy, when compared with land birth. These findings support the use of water birth as a potentially safe option when strict selection criteria are applied within a supervised clinical environment. In the broader context of rising cesarean section rates and increasing efforts to promote physiological childbirth, water birth may represent a valuable strategy for supporting vaginal birth while maintaining maternal and neonatal safety. Further prospective and multicenter studies are warranted to better define its role within modern obstetric care.

## Figures and Tables

**Table 1:** Baseline maternal characteristics by birth cohort.

Variable	Water Birth (*n* = 692)	Land Birth (*n* = 561)	*p*-Value
Maternal age (years), mean ± SD	30.73 ± 4.67	30.20 ± 4.72	0.061
*(95% CI)*	30.38–31.08	29.81–30.59	—
Gestational age (weeks), mean ± SD	39.1 ± 1.1	39.0 ± 1.2	NS
Nulliparous, *n* (%)	~58%	~58%	NS

NS = not statistically significant (*p* > 0.05). Values are expressed as mean ± standard deviation (SD) or percentages.

**Table 2:** Consolidated maternal and neonatal outcomes by birth cohort, with unadjusted and adjusted odds ratios.

Variable	Water Birth (n = 692)	Land Birth (n = 561)	*p*- Value	Crude OR (95% CI)	Adjusted OR * (95% CI)
**MATERNAL OUTCOMES**					
Episiotomy, *n* (%)	35 (5.06%)	77 (13.72%)	<0.001	0.33 (0.22– 0.50)	0.44 (0.29–0.68)
*(95% CI for rate)*	3.5–6.6%	10.8–16.6%	—	—	—
Perineal Tears (Grade I/II), *n* (%)	—	—	<0.001	0.38 (0.22– 0.58)	0.44 (0.29–0.68)
Postpartum Hemorrhage, *n* (%)	—	—	0.320	0.62 (0.34– 1.12)	0.74 (0.41–1.35)
**NEONATAL OUTCOMES**					
Birth weight (g), mean ± SD	3261.7 ± 467.2	3266.4 ± 463.6	0.867	—	—
*(95% CI)*	3226.9–3296.5	3228.1–3304.7	—	—	—
Low birth weight (<2500 g), *n* (%)	—	—	0.860	0.92 (0.38– 2.22)	—
Apgar score (1 min), mean ± SD	9.17 ± 0.97	9.17 ± 0.90	0.971	—	—
*(95% CI)*	9.10–9.24	9.10–9.24	—	—	—
Apgar score < 7 (1 min), *n* (%)	—	—	0.910	0.94 (0.35– 2.53)	—
Apgar score < 9 (1 min), *n* (%)	—	—	0.004	0.28 (0.12– 0.65)	0.36 (0.18–0.72)
NICU Admission, *n* (%)	0 (0%)	5 (0.9%)	0.062 †	0.54 (0.21– 1.38)	0.61 (0.25–1.49)

Values are expressed as mean ± SD or n (%). * Adjusted for maternal age, parity, and gestational age. Fisher’s Exact Test used due to zero-cell frequency. OR = Odds Ratio; CI = Confidence Interval; NICU = Neonatal Intensive Care Unit. A *p*-value < 0.05 was considered statistically significant. † Fisher’s Exact Test used due to zero-cell frequency.

## Data Availability

The datasets generated and analyzed during the current study are not publicly available due to ethical and privacy restrictions involving clinical patient data. However, anonymized data may be made available from the corresponding author upon reasonable request and with permission from the institutional ethics committee.

## Abbreviations

The following abbreviations are used in this manuscript:

NICU	Neonatal Intensive Care Unit
SD	Standard deviation
KDE	Overlapping density curves
PRP	Platelet-Rich Plasma
CI	Confidence Interval
OR	Odds Ratio
